# Rapid Improvement Project: Improving Caregivers’ Understanding of Safety Recommendations for Neurosurgical Devices

**DOI:** 10.1097/pq9.0000000000000376

**Published:** 2020-12-28

**Authors:** Miracle C. Anokwute, Dianne Seibold, Andrew Jea, Laurie L. Ackerman, Jeffrey S. Raskin

**Affiliations:** From the* Section of Pediatric Neurosurgery, Riley Hospital for Children, Indiana University School of Medicine, Department of Neurological Surgery, Indianapolis, Ind.; † Clinical Service Coordinator for Neurosurgery, Riley Hospital for Children, Indiana University Health, Indianapolis, Ind.

## Abstract

There has been a proliferation in the development of indwelling neuromodulatory devices with varied safety recommendations, making it difficult for providers to remain up-to-date. This deficit presents an opportunity for significant improvement in patient safety.

**Methods:**

We performed a search for monopolar electrocautery and magnetic resonance imaging safety recommendations for several indwelling neuromodulatory devices. We developed a questionnaire followed by an educational compendium and a posttest for 50 care providers.

**Results:**

Overall, there was a poor performance on the pretest (mean 39%, SD 19%) but significant improvement on the posttest (mean 71%, SD 16%), *P* < 0.0001. We placed the educational compendium that included all manufacturer recommendations in the operating room for easy reference. A 2.4 times decrease in the case start times of vagus nerve stimulator cases is evidence of its effectiveness.

**Conclusions:**

The authors highlight the lack of knowledge about manufacturer safety recommendations for indwelling neurosurgical devices, which led to the creation of operating room supplements and educational devices.

## Abstract 2

Supplemental Digital Content is available in the text.

## INTRODUCTION

### Problem Description

Since the 1980s, there has been an exponential proliferation in the development and implantation rate of indwelling neurosurgical devices for neuromodulation of many conditions affecting the central nervous system.^1^ In functional neurosurgery, a combination of industry advocacy and improved technology has driven the field away from ablative procedures towards neuromodulation with the implantation of increasingly sophisticated indwelling devices.^2,3^

### Available Knowledge

Today, the implantation of indwelling neurosurgical devices is quite common. For example, in 2012, Cyberonics reported the 100,000th implantation of its Vagus Nerve Stimulator (VNS) systems (Cyberonics, Inc: Houston, Tex.).^4^ Estimates of hydrocephalus are 0.2 to 0.8 per 1,000 live births in the United States for which the standard of treatment is an implanted cerebral spinal fluid shunt.^5,6^ With such high uses of indwelling neurosurgical devices and despite each device having its factory restrictions and recommendations, there is a lack of knowledge among treating practitioners regarding manufacturer monopolar electrocautery and magnetic resonance imaging (MRI) safety recommendations. MRI safety recommendations can be confusing and are usually specific, and devices are defined as either MRI safe, MRI conditional, or MRI unsafe. The resulting nuances among devices contextualized within individual patients are a potential safety issue and represent an opportunity for significant improvement.^7^

### Specific Aims

This study aims to expose the lack of electrocautery and MRI safety knowledge of practitioners and rectify their knowledge gap through education. We aim to increase the practitioners’ knowledge of MRI and electrocautery safety from 39% pretest to 100% posttest after an educational session. Additionally, in VNS patients, we aim to improve the surgical case start delays with a newly implemented protocol in 2 years. Finally, preventable adverse events are easy targets in the goal of improved patient safety. Dedicated device-specific protocols and improved caregiver knowledge should mitigate the risk of preventable device complications from electrocautery and MRI.

## METHODS

We performed an online search for manufacturer recommendations for electrocautery and MRI safety for the following indwelling neurosurgical devices: nonprogrammable shunt valves,^8^ programmable shunt valves,^9–11^ SynchroMed II baclofen pump (Medtronic, Inc., Minneapolis, Minn.),^12^ LivaNova (previously Cyberonics) VNS,^13,14^ Medtronic Intellis spinal cord stimulator (Medtronic, Inc.),^15,16^ Medtronic Deep Brain Stimulator (DBS),^17^ and NeuroPace Responsive Neurostimulator System.^18^ Recommendations from manufacturer’s pamphlets and websites were confirmed with company representatives and condensed into an educational document. Within the MR environment, MRI safe devices do not require considerations, MRI unsafe devices are never safe, and MR conditional devices can be used safely with considerations. Excluded in this study were stabilizing and fixation devices, including screws and rods.

### Measures

Using information gathered from manufacturer recommendations, we developed a 15-point questionnaire, and an educational document describing operative and testing considerations for patients at our tertiary care center with indwelling functional neurosurgical devices (**see Supplemental Digital Content 1,** which demonstrates demographic data collected and questions and shows the answer key (not originally present for subject testing), http://links.lww.com/PQ9/A229 and **Supplemental Digital Content 2,** which Operating Room Compendium and Table of Contents (page 1); and Manufacturer recommendations for institutional indwelling functional neurosurgical devices, http://links.lww.com/PQ9/A230). The pretest was provided to operating room treating practitioners, including nurses and technicians, neurosurgeons and neurologists at different levels of training (ie, attendings, fellows, residents, and students.) After completing the pretest, we immediately provided an educational review of manufacturer safety recommendations based on manufacturer websites for all participants. Finally, we administered a posttest utilizing the same format as the pretest within 24 hours after administering the educational document. Of note, operating room nurses and technicians were tested 1 month after physicians and medical students.

### Analysis

We analyzed categorical data, including pretest and posttest scores, using unpaired students *t* test for the entire cohort. Subgroup analysis of physicians and ancillary practitioners (nurses, technicians, and medical students) of the mean percent score difference was performed via unpaired *t* tests.

**Fig. 1.**
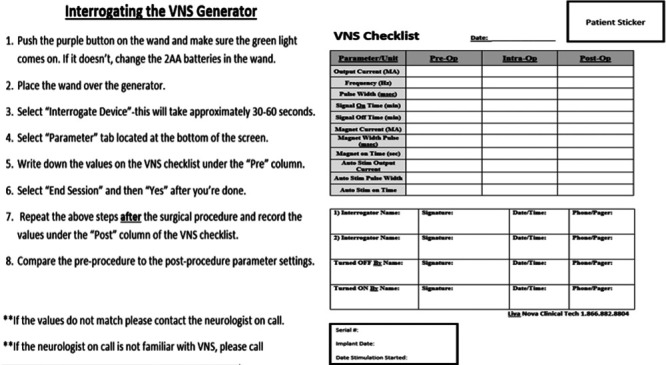
Left, New institutional protocol guiding nurse or neurologist to interrogate the VNS before and following surgery when using monopolar electrocautery. Right, The corresponding checklist.

As a consequence of this rapid improvement project, we created 2 work-products. An operative room compendium was made by collocating manufacturer recommendations for easy reference and placed it in all operating rooms (**see Supplemental Digital Content 1,** which demonstrates demographic data collected and questions and shows the answer key (not originally present for subject testing), http://links.lww.com/PQ9/A229 and **Supplemental Digital Content 2,** which Operating Room Compendium and Table of Contents (page 1); and Manufacturer recommendations for institutional indwelling functional neurosurgical devices, http://links.lww.com/PQ9/A230). We redesigned the VNS (LivaNova, London, England) protocol to include preoperative interrogation but no changes to the output (Fig. 1). To determine the quality improvement study outcomes, we searched unit secretary logs for case delays citing the “VNS,” preimplementation and postimplementation of the revised VNS stimulator protocol.

### Ethical Considerations

This project did not require formal review and approval by the institutional review board. This study falls under a survey study that did not identify the subjects and, therefore, did not identify any ethical issues pertaining to patients with devices or providers.

## RESULTS

We identified 50 care providers who underwent pretesting and posttesting. There were 17 physicians (attending, fellow, or resident), one medical student, 25 operating room nurses, and 6 operating room technicians. Of the physicians, 41% were neurosurgeons, 41% were neurologists, and 18% chose not to identify their specialty. The overall cohort included 50 respondents with poor performance on the pretest with a mean score of 39% (SD 19%) but statistically significant improvement to a mean score of 71% (SD 16%), *P* < 0.0001 on the posttest.

Physicians had a poor pretest performance (mean 51%, SD 21%) but improved on the posttest (mean 73%, SD 12%), *P* = 0.0007. However, when analyzing attending physicians alone, although there is poor pretest performance, the attending subgroup demonstrated the highest pretest score (mean 61%, SD 24%). They did not demonstrate statistically significant improvement on the posttest (mean 72%, SD 17%), *P* = 0.36. Residents and fellows had a far worse pretest performance than attending physicians (mean 43%, SD 5%) but significantly improved posttest (mean 74%, SD 3%), *P* = 0.0001.

**Fig. 2.**
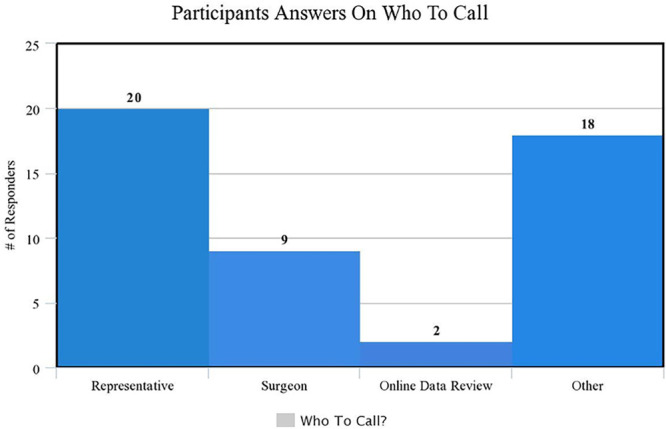
Histogram demonstrating the majority of participants would call the representative of the device manufacturer when safety recommendations were not known.

Findings were similar in a subgroup analysis of ancillary practitioners (nurses, students, and techs) with a pretest mean of 33% (SD 14%) and a posttest mean of 71% (SD 17%), *P* = 0.001. Notably, although both cohorts improved after the educational teaching session, the ancillary practitioners had the most significant pretest and posttest mean score difference at 38% (SD 23%) compared to physicians’ 22% difference (SD 17%), *P* = 0.017. When asked who should be called or asked when determining manufacturer safety recommendations, most respondents (41%) called the manufacturer representative (Fig. 2).

A retrospective search of unit secretary logs returned four preimplementation delays out of 1,296 cases (0.31%) in patients with a VNS device in the 2 years before implementation. After implementing our new protocol and safety compendium, only one delay occurred among 1135 VNS cases (0.09%), demonstrating 2.4 times improvement in VNS case delays. The absolute number of surgical cases experiencing a delay in start time decreased considerably after implementing our protocol/education session. Although it did not reach statistical significance, there was a trend toward a meaningful difference between the preprotocol and postprotocol groups.

## DISCUSSION

### Summary

Although practitioners use indwelling functional neurosurgical devices to treat a wide variety of neurosurgical disorders, we have uncovered a profound knowledge gap among practitioners regarding the manufacture safety recommendations of these devices in the setting of MRI and monopolar electrocautery. This lack of understanding impacts neurosurgeons, who should be familiar with these devices, but also involves other physicians who order diagnostic testing or perform operative procedures on these patients without being aware of the limitations the devices impose. This knowledge deficit is a potentially preventable patient safety issue, and improvements must be made to rectify this knowledge gap among practitioners. Therefore, we developed a 15-point questionnaire to highlight this issue among practitioners in our institution and an educational compendium with an informational lecture to rectify this knowledge gap (**see Supplemental Digital Content 1,** which demonstrates demographic data collected and questions and shows the answer key (not originally present for subject testing), http://links.lww.com/PQ9/A229).

The authors found a significant deficiency of knowledge on safety recommendations for functional indwelling neurosurgical devices with an overall mean score of 39% on the pretest. Physicians who implant and regulate these devices scored slightly above the overall cohort average with a mean score of 51%. Similar findings were noted for the ancillary practitioner subgroup that included nurses, medical students, and technicians with a 33% mean pretest score. Although attending physicians had a knowledge gap, they outscored all subgroups in the pretest portion of this study. Knowing whether a device is MRI or electrocautery conditional or safe is essential to establish before the procedure and is quintessential to patient safety. For example, the manufacturer’s recommendations for monopolar electrocautery safety with LivaNova VNS system indicate that electrocautery may be used in a patient with an already implanted VNS system but should not be utilized during the initial implantation of the system as this may damage not only the VNS system but also the Vagus nerve and other structures within the carotid sheath.^13^

### Interpretation

When faced with a device for which manufacturer safety settings were unknown, most respondents selected that they would call the manufacturer representative. However, our study demonstrates that even when practitioners are sure of the manufacturer’s safety settings, they are more likely to be incorrect, as seen by the dismal pretest performance. We can rectify this lack of knowledge with appropriate teaching. However, the manufacturers’ guidelines should always be consulted before initiating a procedure or MRI scan as the manufacturers’ recommendations are the most up-to-date information source.

Through performing this patient safety study, we uncovered and subsequently addressed several potential problems at our institution. After a short educational session over manufacturer safety settings for the above devices, there was a statistically significant improvement in the overall cohort and each subgroup’s mean percent score. Additionally, an educational document was provided for participants in the study and subsequently printed for real-time use in the operating rooms.

As a result of this study, we have reviewed and implemented changes to our institutional protocols for indwelling functional neurosurgical devices. Nonprogrammable shunt valve settings do not require verification following MRI based on manufacturer recommendations, and there are no restrictions on monopolar electrocautery. There are no prohibitions on monopolar electrocautery for programmable shunt valves; however, programmable shunt valves are MRI conditional due to the system’s magnetic components. The valve settings must be verified after MRI.

At our institution, we evaluate implanted Synchromed II pumps following MRI to ensure continued appropriate function. We use monopolar electrocautery in patients with previously implanted VNS systems without turning the device off. However, we have a neurologist or a nurse to evaluate the VNS system preoperatively and again postoperatively to verify the VNS setting (Fig. 1). We do not use monopolar electrocautery when implanting the VNS system after the generator is placed on the sterile field. We exclude the area of the implant (C7-T8) from the radiofrequency field during MRI with implanted VNS in line with LivaNova guidelines. If broken electrodes are encountered, we use the transmit/receive head or extremity coils only, and never deploy the transmit/receive body coils according to LivaNova guidelines. Due to the variability in the VNS systems’ components and the anatomical variations for depending on the MRI scan, the device is considered MR conditional.

At our institution, all patients with Medtronic Intellis spinal cord stimulators must have their device verified as safe on the Medtronic MRI checklist and must be placed in MRI safe mode by the Medtronic representative. The device is MRI conditional, with MRI safety conferred by following this proprietary recommendation. As Medtronic provides no guarantee that monopolar electrocautery is safe even if used with restrictions, surgeons may use monopolar electrocautery based on preference and liability.

For patients with indwelling Medtronic DBS systems, the system is verified with the Medtronic MRI manual to determine MRI safety. If the device is MRI safe or conditional based on the MRI manual, switch off the device, and use the transmit/receive body or head coils. Monopolar electrocautery is not safe but may be used at the discretion of the surgeon if the DBS device is off and the current flow is perpendicular from a line demarcating leads from the generator.^17^

Due to the contraindications against MRI and monopolar electrocautery for the NeuroPace Responsive Neurostimulator system, patients with this system are prohibited from undergoing MRI^19^ and using monopolar electrocautery during surgery.

## LIMITATIONS

This study demonstrates a lack of knowledge among practitioners and provides suggestions for bridging that knowledge gap, hopefully improving patient safety; however, there are several shortcomings. A critical reader might posit that the collected manufacturer recommendations are freely available on the internet and that this collocation of recommendations is superfluous. We disagree that the recommendations are easy to find, especially because such an investigation prevents patients from coming to the operating room on time. Although the risk to patient safety is potentially catastrophic (eg, carotid sheath injury and thermal spinal cord injury), the number of potential near misses is unquantifiable because we do not prospectively record surgical procedures on patients with indwelling neurosurgical devices. This study was a test in short-term memory after providing an educational, informational session. Long-term follow-up is needed to elucidate if the education provided is appropriately utilized in patient care. We believe this type of information may need to be reviewed annually. It would suggest the development of robust preoperative and pre-MRI screening protocols to identify the device’s presence and alert personnel to possible implications for planned procedures. This alert should be augmented with easy access to an informational resource such as was developed for this project (**see Supplemental Digital Content 2,** which Operating Room Compendium and Table of Contents (page 1); and Manufacturer recommendations for institutional indwelling functional neurosurgical devices, http://links.lww.com/PQ9/A230).

The authors did not explore patient/family member knowledge of the device and its implications in MRI or operative settings. As not all patients may undergo care at a tertiary facility familiar with the device, this is an area that would benefit from further study. Last, this is a single-center study; a multicenter study would further demonstrate deficiencies in the knowledge base of manufacturer safety recommendations for indwelling neurosurgical devices nationally and allow for collaborative approaches to improve patient safety. Since we presented this work at the 2019 joint annual American Association of Neurological Surgeons/Congress of Neurological Surgeons pediatric section meeting, 2 other large academic centers have implemented this project, and a multisite quality improvement project is being discussed. Due to the rapid proliferation of indwelling devices and the changing of existing safety recommendations, national attention to proprietary recommendations should be centralized. The authors recommend reviewing the specific safety recommendations for each device within the particular clinical context before performing electrocautery or MRI.

## CONCLUSIONS

Overall, this study emphasizes and highlights the significant lack of knowledge about the manufacturer safety recommendations for indwelling functional neurosurgical devices at a single institution. Ongoing education is critical to improving patients’ safety with these devices, with suggestions for developing accessible protocols. Studying the potential for preventable patient safety events has changed the VNS interrogation protocol leading to a 39% decrease in case start delays and caused the development of collocated factory recommendations to improve patient safety in neurosurgery. In the future, we plan to expand this study and provide the device compendium to other institutions, as this is not an isolated problem.

## DISCLOSURE

The authors have no financial interest to declare in relation to the content of this article.

## ACKNOWLEDGMENTS

The authors thank Glenda Shaw for her editorial assistance.

## Supplementary Material


