# Supplementary material for: Rapid Improvement Project: Improving Caregivers’ Understanding of Safety Recommendations for Neurosurgical Devices
Source: Pediatr Qual Saf. 2020 Dec 28;6(1):e376. doi: 10.1097/pq9.0000000000000376 (PMC7781352; doi:10.1097/pq9.0000000000000376)
Supplement: Supplementary file 1 [file pqs-6-e376-s001.pdf]

SUPPLEMENTAL DIGITAL CONTENT 1

In-dwelling Neurosurgical Medical Device Questionnaire

Credentials (circle one):

Attending Fellow Resident Nurse Technician Other

Purpose: Neurosurgical patients often have indwelling devices which may limit imaging and surgical procedures. Practitioners involved in the surgical management of patients with these devices should be aware of associated safety recommendations.

Please select one answer which modifies the question stem most appropriately.

Monopolar electrocautery (Bovie) is safe to use in patients with indwelling nonprogrammable shunt valves.

A. Yes, monopolar electrocautery can be used without restriction.
B. Yes, monopolar electrocautery can be used but not on the device itself.
C. No, monopolar electrocautery cannot be used because it causes device malfunction.
D. No, it is never appropriate to use monopolar electrocautery because it causes tissue injury around the device.

Patients with indwelling nonprogrammable shunts are safe to undergo magnetic resonance imaging (MRI).

A. Yes, MRI is safe and does not affect the shunt setting.
B. Yes, MRI is safe but it the setting needs to be checked afterwards.
C. No, MRI is unsafe and causes irreversible damage to the valve.
D. No, MRI is unsafe and causes local tissue damage.

Patients with indwelling programmable shunts including the Codman and Medtronic Strata valves are safe to undergo magnetic resonance imaging (MRI).

A. Yes, MRI is safe and does not affect the shunt setting.
B. Yes, MRI is safe but it the setting needs to be checked afterwards.
C. No, MRI is unsafe and causes irreversible damage to the valve.
D. No, MRI is unsafe and causes local tissue damage.

Monopolar electrocautery (Bovie) is safe to use in patients with indwelling programmable shunts including the Codman and Medtronic Strata valves.

A. Yes, monopolar electrocautery can be used without restriction.
B. Yes, monopolar electrocautery can be used but not on the device itself.
C. No, monopolar electrocautery cannot be used because it causes device malfunction.
D. No, it is never appropriate to use monopolar electrocautery because it causes tissue injury around the device.

Patients with an indwelling Medtronic SynchroMed II pump (baclofen pump) are able to undergo magnetic resonance imaging (MRI).

A. Yes, MRI is safe and does not affect the Medtronic SynchroMed II pump.
B. Yes, MRI is safe, but it suspends drug infusion, requiring the functionality to be checked afterwards.
C. No, MRI is unsafe and causes irreversible system damage to the Medtronic Synchromed II pump.
D. No, MRI is unsafe and causes local tissue damage.

Patients with an indwelling Cyberonics Vagus Nerve Stimulator (VNS) and intact electrodes are safe to undergo magnetic resonance imaging (MRI).

A. Yes, MRI is safe and does not affect the VNS.
B. Yes, MRI is safe if the area of the implant (C7-T8) is excluded from the radiofrequency field.
C. No, MRI is unsafe and causes irreversible damage to the VNS.
D. No, MRI is unsafe and causes damage to the Vagus nerve.

Patients with an indwelling Cyberonics Vagus Nerve Stimulator (VNS) and either intact or suspected broken electrodes are safe to undergo magnetic resonance imaging (MRI).

A. Yes, MRI is safe and does not affect the VNS, but only in 1.5T strength.
B. Yes, MRI is safe in 1.5 and 3T strength, but the transmit/receive head or extremity coils must be used.
C. No, MRI is unsafe unless the patient magnet is taped over the device.
D. No, MRI is unsafe and causes irreversible damage to the VNS.

Patients with an indwelling Cyberonics Vagus Nerve Stimulator (VNS) and either intact or suspected broken electrodes are safe to undergo magnetic resonance imaging (MRI).

A. Yes, MRI is safe when the magnet is taped over the device.
B. Yes, MRI is safe if the output current parameters are set to zero.
C. No, MRI is unsafe unless the entire device is “turned off.”
D. No, MRI is unsafe and causes irreversible damage to the VNS.

Monopolar electrocautery (Bovie) is safe to use in patients who have an indwelling Cyberonics Vagus Nerve Stimulator (VNS).

A. Yes, monopolar electrocautery can be used without restriction.
B. Yes, monopolar electrocautery is safe but current flow through the generator and leads must be minimized and the device checked following surgery.
C. No, monopolar electrocautery cannot be used within 30cm of an implanted system.
D. No, both monopolar electrocautery is a factory contraindication when a patient has a VNS.

Patients with an indwelling Medtronic Intellis™ spinal cord stimulator (SCS) and intact electrodes are safe to undergo magnetic resonance imaging (MRI).

A. Yes, but only if specified by the Medtronic Intellis MRI eligibility check-list and device is in MRI mode.
B. Yes, MRI is safe if the device stimulation is turned off and the device is in MRI mode.
C. No, MRI is unsafe unless the entire device is “turned off.”
D. No, MRI is unsafe and causes irreversible damage to the SCS.

Monopolar electrocautery (Bovie) is safe to use in patients who have an indwelling Medtronic Intellis spinal cord stimulator (SCS).

A. Yes, monopolar electrocautery can be used without restriction.
B. Yes, there is a guarantee that both monopolar electrocautery can be used within 15cm of the SCS.
C. Yes, monopolar electrocautery can be used within 30cm of the SCS.
D. There is no guarantee that monopolar electrocautery is safe even if used with restrictions.

Patients with an indwelling Medtronic Deep Brain Stimulator (DBS) and intact electrodes are safe to undergo magnetic resonance imaging (MRI).

A. Yes, MRI is safe when the magnet is taped over the device.
B. Yes, MRI is safe if the device is turned off and the transmit/receive coil is used.
C. No, MRI is unsafe unless the generator is removed.
D. No, in general MRI is contraindicated as it damages the generator.

Monopolar electrocautery (Bovie) is safe to use in patients who have an indwelling Medtronic DBS and electrodes.

A. Yes, monopolar electrocautery can be used within 15cm of the DBS.
B. Yes, monopolar electrocautery can be used within 30cm of the DBS.
C. No, monopolar electrocautery is not safe but can be used if the device is off and current flow is perpendicular from a line demarcating leads from generator.
D. No, monopolar electrocautery is a factory contraindication when a patient has a DBS.

Patients with an indwelling Responsive Neurostimulator System (RNS®) and intact electrodes are safe to undergo magnetic resonance imaging (MRI).

A. Yes, MRI is safe when the magnet is taped over the device.
B. Yes, MRI is safe if the device is turned off and the transmit/receive coil is used.
C. No, MRI is unsafe unless the generator is removed.
D. No, in MRI is contraindicated as the system is MR Unsafe.

Monopolar electrocautery (Bovie) is safe to use in patients who have an indwelling Responsive Neurostimulator (RNS) and electrodes.

A. Yes, monopolar electrocautery can be used without restriction.
B. Yes, monopolar electrocautery can be used within 15cm of the RNS.
C. Yes, monopolar electrocautery can be used within 30cm of the RNS.
D. No, monopolar electrocautery is a factory contraindication when a patient has a RNS.

Answer sheet:

1. A 2. A 3. B 4. A 5. B 6. B 7. B 8. B 9. B 10. A 11. D 12. B 13. C 14. D 15. D

Legend: Survey demonstrates demographic data collected, questions, and shows answer key (not originally present for subject testing).
