# Supplementary material for: Rapid Improvement Project: Improving Caregivers’ Understanding of Safety Recommendations for Neurosurgical Devices
Source: Pediatr Qual Saf. 2020 Dec 28;6(1):e376. doi: 10.1097/pq9.0000000000000376 (PMC7781352; doi:10.1097/pq9.0000000000000376)
Supplement: Supplementary file 2 [file pqs-6-e376-s002.pdf]

SUPPLEMENTAL DIGITAL CONTENT 2

Operating Room Indwelling Neurosurgical Device Safety Compendium

Table of Contents

Terms**:**

Shunt Valves
Nonprogrammable
    Bovie
    MRI
Programmable
    Bovie
    MRI

Baclofen Pump
    Bovie
    MRI

Vagus Nerve Stimulation:
    Bovie
    MRI

Spinal Cord Stimulation
    Bovie
    MRI

Deep Brain Stimulation
    Bovie
    MRI

Responsive Neurostimulation:
    Bovie
    MRI

References

Terms:
Monopolar Electrocautery: Commonly known in the OR as “Bovie.”

Manufacturer recommendations for institutional indwelling functional neurosurgical devices

Shunt valves

Electrocautery (bovie) for nonprogrammable shunt valves
There is no advisory against or for use of electrocautery for non-programmable shunt valves

Recommendation
Monopolar electrocautery can be used without restriction.

MRI for non-programmable shunt valves
MRI is safe for non-programmable shunt valves

Recommendation
MRI is safe and does not affect the shunt setting.

Electrocautery (bovie) for programmable shunt valves:
There is no advisory against or for use of electrocautery for programmable shunt valves.

Recommendation
Monopolar electrocautery can be used without restriction.

MRI for programmable shunt valves

Strata:
The strata II valve is classified as MRI conditional. Per Medtronic, the Strata valve mechanism will not be damaged in MRI systems up to 3 tesla. However, the performance level setting can change. Therefore, the shunt settings should always be checked before and after exposure to the MRI magnet.

Codman Certas:
The Codman programmable valve (Certas) is classified as MRI conditional. MRI may be performed at anytime after implantation if the MRI systems has a static magnetic field of 3 tesla or less and a spatial gradient of 720Guass/cm or less. However, the shunt valve setting must be verified after exposure to the MRI magnet.

Recommendation
MRI is safe but it the setting needs to be checked afterwards.

Baclofen Pump

Monopolar (bovie) SynchroMed II

Monopolar (bovie) electrocautery can be utilized on synchromed II devices without restrictions

Recommendation:
Monopolar electrocautery can be used without restriction.

MRI SynchroMed II

MRI may be utilized in patients with the SynchroMed II as it is MRI conditional. However, temporary motor stall and stall recovery may occur. Although the pump should technically resume normal operation upon MRI completion, it may take up to 2 to 24hrs for pump to return to appropriate infusion after MRI completion.

Recommendation
MRI is safe, but it suspends drug infusion, requiring the functionality to be checked afterwards.

Vagus Nerve Stimulation (VNS)

Monopolar (bovie) Cyberonics VNS

Caution must be taken with use of electrocautery in patients with Cyberonics VNS leads and generators. When implanting Cyberonics VNS, electrocautery should not be utilized after the generator is placed in the sterile field. Electrocautery may be used in a patient who already has an implanted Cyberonics VNS generator, but care should be taken to minimize the current flowing through the generator and lead system. Cyberonics suggests positioning the electrosurgical electrodes as far as possible from the generator and lead, avoiding electrode placement in direct path of current flow to the lead or generator or in the same body cavity as the generator, and confirming the generator is functioning as programmed after electrosurgery.

Recommendation

When implanting Cyberonics VNS, electrocautery should not be utilized after the generator is placed in the sterile field. Electrocautery may be used in a patient who already has an implanted Cyberonics VNS generator, but care should be taken to minimize the current flowing through the generator and lead system.

MRI Cyberonics VNS intact Electrodes

Cyberonics VNS with intact electrodes are considered MRI conditional if Cyberonics guidelines are followed. If Cyberonics guidelines are not adhered to including exclusion of the area of the implant from the radiofrequency field, there is a risk for heating of the lead electrodes, which can result in temporary injury, necrosis, or permanent tissue damage including damage to the vagus nerve and structures of the carotid sheath.

Recommendation
MRI is safe if the area of the implant (C7-T8) is excluded from the radiofrequency field.

MRI Cyberonics VNS broken electrodes

Care should be taken with MRI's on patients with suspected or known broken leads as the exposed lead wire is a point at risk for heating of the electrode leading to damage above. These are still MRI conditional in 1.5 and 3 tesla MRI's but only if the VNS current parameters are set to zero and the transmit/receive head or extremity coils as far as the > 2cm of the lead is still implanted and <2cm is exposed. The transmit/receive body coil must never be used in these patients. If a transmit RF body coil is needed then surgery must be undertaken to remove the VNS system prior to imaging.

Recommendation
MRI is safe in 1.5 and 3T strength, but the transmit/receive head or extremity coils must be used.

Spinal Cord Stimulation

Electrocautery (bovie) Medtronic Intellis™ Spinal Cord Stimulator and Intact Electrodes

Electrocautery may transmit electromagnetic interference which may lead to patient injury or death, damage to the neurostimulation system and damage to the surrounding tissue. Although the neurostimulator contains features that protect from electromagnetic interference this is not a guarantee.

Recommendation
There is no guarantee that monopolar electrocautery is safe even if used with restrictions.

MRI Medtronic Intellis Spinal Cord Stimulator and Intact Electrodes

Medtronic Intellis Spinal Cord Stimulator and electrodes are considered MRI conditional. An MRI scan should never be done unless the implanted model is known and determined to be safe as only certain models can undergo MRI scanning.

Recommendation
MRI safe only if specified by the Medtronic Intellis MRI eligibility check-list and device is in MRI mode.

Deep Brain Stimulation (DBS)

Electrocautery (bovie) Medtronic DBS

It is recommended that electrocautery be avoided at all times for patients with Medtronic DBS system as use of electrocautery can result in insulation damage around the lead or lead extension, component failure, or induced currents into the patient leading to tissue damage, patient stimulation or patient shock. Additionally, damage to the neurostimulator can lead to suppressed or increased stimulation. However, if electrocautery must be used, it should be used with the following precautions: the neurostimulator must be turned off prior to use and the cable

connecting the lead or extension to a screener or external neurostimulator must be disconnected. Additionally, only bipolar cautery should be utilized but if unipolar cautery is necessary then only use the low-voltage mode at the lowest possible power setting with the current path or ground plate as far from the neurostimulator, extension, and lead as possible. The full-length operating room table grounding pad should not be used, and the electrocautery current flow should be perpendicular to a line drawn between the neurostimulator case and lead electrodes. Neurostimulator function should be determined after using electrocautery.

Recommendation
Monopolar electrocautery is not safe but can be used if the device is off and current flow is perpendicular from a line demarcating leads from generator.

MRI for Medtronic DBS

Medtronic DBS system is considered MRI conditional as some models can lead to tissue heating, permanent injury, coma, paralysis, or death if Clinicians should refer to the Medtronic MRI manual to determine which systems are MRI compatible. For those found to be compatible, MRI's may be performed only with body or head transmit/receive RF coils.

Recommendation
MRI is safe if the device is turned off and the transmit/receive coil is used.

Responsive Neurostimulation System (RNS)

RNS NeuroPace® Electrocautery (bovie)
Electrocautery is contraindicated in the RNS NeuroPace system.

Recommendation
Monopolar electrocautery is a factory contraindication when a patient has an RNS.

RNS NeuroPace MRI
MRI is contraindicated in patients with the RNS NeuroPace system and is classified as MRI Unsafe.

Recommendation
MRI is contraindicated as the system is MR Unsafe.

References:
[As appears in manuscript]

9. Codman Hakim Programmable Valve System for Hydrocephalus: Procedure Guide. 2006

10. Codman Certas Plus Programmable Valve. Codman Neuro. Deputy Synthes. 2015

11. Medtronic Strata II Valve: Instructions for Use. https://www.medtronic.com/us-en/healthcare-professionals/products/neurological/shunts/strata-adjustable-pressure-valve/manuals-technical-resources.html. Accessed: January 24, 2019.

12. Medtronic SynchroMed IsoMed 8626, 8627, 8637, 8472 Implantable Infusion Systems: Information for Prescribers (Includes MRI Eligibility Information). 2017

13. VNS Therapy System Physicians Manual. 2018

14. MRI With the VNS Therapy System. 2017

15. Medtronic Pain Therapy Using Intellis Neurostimulation System for Chronic Pain. Information for Prescribers. 2017

16. MRI Guidelines for Medtronic Neurostimulation Systems for Chronic Pain: Instructions for Use. 2017.

17. MRI Guidelines for Medtronic Deep Brain Stimulation Systems: Instructions for Use. 2015.

18. Medtronic DBS Therapy Implanted Neurostimulators: Information for Prescribers. 2017.

19. NeuroPace. NeuroPace® RNS® System Brief Statement. http://www.neuropace.com/safety/. Accessed: January 24, 2019.
